# Themes of Reported Rural Healthcare Challenges Related to Non-Communicable Diseases Before and After the COVID-19 Pandemic: A Scoping Review

**DOI:** 10.3390/ijerph23080971

**Published:** 2026-07-27

**Authors:** Takashi Kuwayama, Kazuhiko Kotani

**Affiliations:** Center for Community Medicine, Jichi Medical University, 3311-1 Yakushiji, Shimotsuke-City 329-0498, Tochigi, Japan; tak_kuwa@mac.com

**Keywords:** rural health, non-communicable diseases, health disparities, social determinants, digital health, COVID-19

## Abstract

**Highlights:**

**Public health relevance—How does this work relate to a public health issue?**

- Rural populations experience persistent disparities in non-communicable disease (NCD) burden and its related management relative to urban areas.
- The COVID-19 pandemic may have influenced the themes of NCD-related challenges in rural areas.

**Public health significance—Why is this work of significance to public health?**

- This review identified themes of reported rural healthcare challenges related to NCDs before and after the COVID-19 pandemic.
- The findings may indicate the growing importance of multimorbidity, social determinants, and digital health in rural areas.

**Public health implications—What are the key implications or messages for practitioners, policy makers and/or researchers in public health?**

- NCD strategies in rural areas might need to move beyond healthcare to include community-based and societal approaches.
- Approaches that utilize digital technologies and resources unique to rural areas may have the potential to mitigate rural health disparities.

**Abstract:**

Non-communicable diseases (NCDs), including cardiovascular disease, cancer, and chronic respiratory disease, are major causes of death worldwide; addressing NCDs is thus essential. NCDs may contribute to health disparities between rural and urban areas. The COVID-19 pandemic has altered medical and social environments, and NCD management approaches may have changed accordingly. This scoping review aimed to examine themes of reported rural healthcare challenges related to NCD management before and after the COVID-19 pandemic. A literature search of original articles was conducted via PubMed. A thematic analysis was applied to compare the content of studies classified as pre-pandemic and post-pandemic. A total of 23 articles were identified, including 12 studies classified as pre-pandemic and 11 studies classified as post-pandemic. Before the pandemic, commonly reported challenges included low management rates of hypertension and diabetes, instability in pharmaceutical supply, and insufficient training of healthcare personnel. We integrated these into a theme related to “healthcare”. After the pandemic, in addition to the low management rates, studies increasingly addressed mental and nutritional health, multimorbidity, and socioeconomic disparities. We integrated there into a theme related to “society”. Namely, studies classified as pre-pandemic primarily reported healthcare-related aspects, whereas studies classified as post-pandemic more frequently reported broader societal aspects in addition to healthcare-related aspects. These findings may reflect changes in how rural healthcare challenges related to NCDs have been recognized and discussed following the COVID-19 pandemic. Further research is needed to clarify the significance and implications of these reported themes.

## 1. Introduction

Non-communicable diseases (NCDs), including cardiovascular disease, cancer, chronic respiratory disease, hypertension, and diabetes, are chronic conditions and major causes of death worldwide [1,2]. Addressing NCDs, regardless of the region, is essential for achieving the Sustainable Development Goals and Universal Health Coverage [3,4]. In rural areas, where healthcare resources and access are generally limited, NCDs can contribute to health disparities compared to urban areas [5]. Therefore, rural healthcare systems must address NCDs [5]. In addition, broader social determinants of health, including socioeconomic conditions, education, transportation, culture, and community support, may influence NCD management in rural areas [6].

The global COVID-19 pandemic, which began at the end of 2019, has altered both medical and social environments worldwide [7]. Previous studies have suggested that the pandemic influenced healthcare access, continuity of health services, and broader social conditions relevant to NCD prevention and management [8,9,10]. These changes may have been particularly important in rural areas, as healthcare resources and access are limited. It would be assumed that these changes have also affected NCD management in rural areas.

Persistent challenges related to NCDs in rural areas have been reported across diverse settings, including limited healthcare access, shortages of healthcare professionals, and constraints in healthcare delivery [11,12,13,14]. In addition, several studies have suggested that the COVID-19 pandemic affected healthcare access, disease control, and related conditions in rural areas [15,16,17]. However, it remains unclear what themes of rural healthcare challenges related to NCDs have been reported with the changes by the COVID-19 pandemic.

The research question of this study was: “What rural healthcare challenges related to NCDs have been explicitly reported before and after the COVID-19 pandemic?” The sub-questions were: (1) What types of rural healthcare challenges related to NCDs have been reported? (2) What approaches or responses have been proposed to address these challenges?, and (3) What themes emerge from the reported challenges and responses?

This review aimed to examine the themes of reported rural healthcare challenges related to NCD management before and after the COVID-19 pandemic. A scoping review was considered appropriate for this objective because the available literature is thought to be heterogeneous in terms of diseases, countries, study designs, and reported challenges. By focusing on literature discussing rural healthcare challenges, this study sought to identify recurring themes that could be useful for future planning of NCD management strategies in rural areas.

## 2. Materials and Methods

### 2.1. Study Design

This scoping review was conducted in accordance with the methodological guidance for scoping reviews proposed by the Joanna Briggs Institute and reported following the Preferred Reporting Items for Systematic Reviews and Meta-Analyses(PRISMA) Extension for Scoping Reviews [18,19,20]. The checklist is provided in Supplementary Materials. The eligibility criteria were defined using the Participants–Concept–Context (PCC) framework recommended for scoping reviews. The participants included rural populations, rural communities, healthcare providers, healthcare systems, or patients living in rural areas. The concept was rural healthcare challenges related to NCDs, including prevention, diagnosis, treatment, management, healthcare access, healthcare workforce, medicine availability, multimorbidity, digital health, health disparities, and social determinants of health. The context was the periods before and after the COVID-19 pandemic.

### 2.2. Search Strategy and Study Selection

A literature search was conducted using PubMed. The search strategy was intentionally designed to identify publications explicitly describing rural healthcare challenges related to NCDs. Therefore, the search included the terms “challenge” and/or “issue” as the primary concepts of interest, because these terms are often used interchangeably in the literature. In addition, the term “rural” was restricted to the title field to ensure that rural healthcare represented the primary focus of the publication rather than a secondary characteristic.

The following search strategy was applied:

(“rural”[Title]) AND (“health”[Title/Abstract]) AND ((“challenge”[Title/Abstract]) OR (“issue”[Title/Abstract])) AND (“non-communicable diseases”[Title/Abstract]).

**Figure 1:**
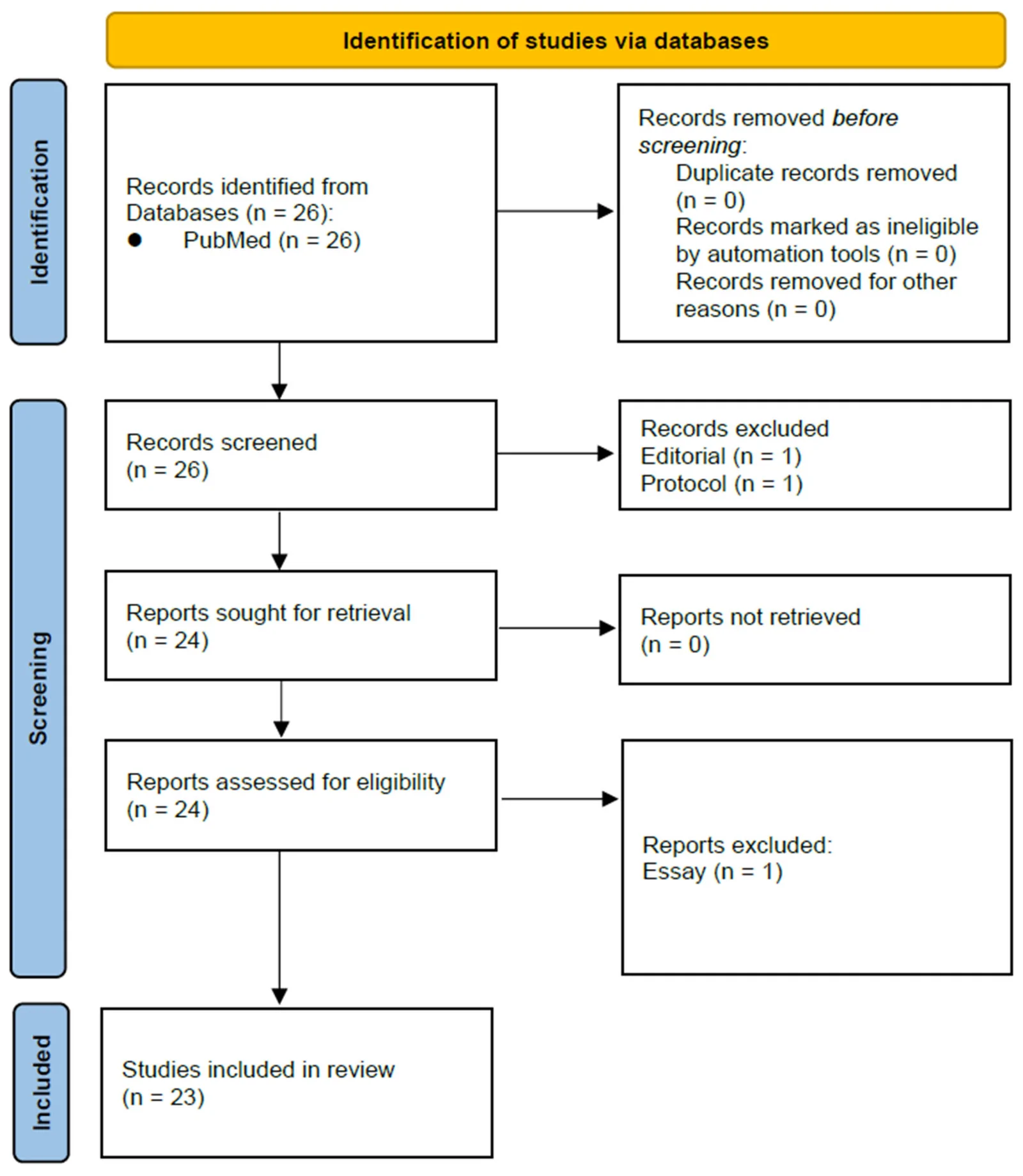
Preferred Reporting Items for Systematic Reviews and Meta-Analyses (PRISMA) flow diagram of article selection.

The final search was conducted on 20 June 2026. Only original articles written in English were included. Letters, review articles, opinion papers, editorials, and conference abstracts were excluded. Titles and abstracts identified through the search were screened according to the eligibility criteria. Studies were included when they explicitly addressed rural healthcare challenges or issues related to NCDs. Articles focusing on NCDs without discussing healthcare challenges or issues were excluded. The selection process is presented in the PRISMA flow diagram (Figure 1).

### 2.3. Classification According to COVID-19 Context

Studies were initially grouped according to publication period (published on or before 31 December 2020 versus published on or after 1 January 2021). To improve classification accuracy, study period, data collection period, submission dates, and explicit discussion of COVID-19-related effects were additionally reviewed whenever available. Final classification into pre-pandemic and post-pandemic groups was based on the best available evidence from these sources.

Data were extracted from the titles and abstracts of the included articles. The extracted information included author and publication year, country, NCD condition, study objective, reported healthcare challenges or issues, reported approaches or responses, study period or data collection period (when available), and explicit discussion of COVID-19-related effects. The extracted information was summarized in Tables 1 and 2.

The timing of publication did not necessarily correspond to the timing of study conduct or data collection. The findings related to temporal changes were interpreted and classified cautiously.

### 2.4. Thematic Analysis

The titles and abstracts of all included articles were carefully examined, and descriptions of the challenges and corresponding approaches related to NCDs in rural areas were extracted. The contents of studies classified as pre-pandemic and post-pandemic were examined. A thematic analysis was applied to identify and organize recurring patterns in the literature [21].

First, reported challenges were extracted from each study. Second, similar concepts were grouped together through iterative review. Third, broader themes were developed by integrating related concepts. Finally, themes identified among pre-pandemic and post-pandemic studies were described and summarized.

The thematic analysis focused on how reported rural healthcare challenges related to NCDs were represented in the literature rather than on evaluating causal effects of the COVID-19 pandemic.

## 3. Results

### 3.1. Study Selection and Characteristics

A total of 23 articles were identified. Based on the best available evidence, including publication period, study period, data collection period, submission dates, and explicit discussion of COVID-19-related effects whenever available, 12 studies were classified as pre-pandemic and 11 studies were classified as post-pandemic (Figure 1; Table 1 and Table 2).

### 3.2. Reported Rural Healthcare Challenges Related to NCDs Before the COVID-19 Pandemic

Among the studies classified as pre-pandemic [11,12,13,22,23,24,25,26,27,28,29,30], several recurring challenges have been reported. These include an increased prevalence and risk of NCDs, such as hypertension and diabetes, accompanied by low management rates. In addition, insufficient education, training, and trust relationships among healthcare workers were noted, as well as instability in the pharmaceutical supply. In response to these challenges, the literature has proposed the development of systems and frameworks for prevention and treatment of NCDs, expansion of healthcare worker education, and establishment of pharmaceutical supply systems through collaboration between governments and non-governmental organizations.

From these findings, key concepts such as lack of healthcare access, insufficient healthcare workforce and education, vulnerability of supply systems, and the need for system development and educational expansion were identified. These were integrated and interpreted as representing a theme focused on “healthcare.”

### 3.3. Reported Rural Healthcare Challenges Related to NCDs After the COVID-19 Pandemic

Among the studies classified as post-pandemic [31,32,33,34,35,36,37,38,39,40,41], similar challenges related to the increasing prevalence and risk of NCDs and low management rates continued to be observed. However, additional challenges have been reported. These included the coexistence of multiple conditions involving NCDs, mental health, nutritional health, and other diseases; health inequalities associated with socioeconomic disparities and ethnic or cultural practices; and lack of a framework for rural health research. In response to these challenges, the literature suggests integrated community health models incorporating mental and nutritional health, early preventive interventions for NCDs, approaches tailored to cultural and individual characteristics, digital interventions such as mobile health, low-cost patient education programs, and the evaluation and standardization of care quality. Key concepts extracted from these studies included multimorbidity and complexity, diversity in cultural and behavioral characteristics, integrated care and comprehensive management, community participation, and the use of digital technology. These were integrated and interpreted as representing a theme focused on “society.”

### 3.4. Themes Identified Through Thematic Analysis

Thematic analysis identified two overarching themes. Among studies classified as pre-pandemic, reported challenges were integrated and interpreted as representing a theme focused on “healthcare.” In contrast, among studies classified as post-pandemic, reported challenges were integrated and interpreted as representing a theme focused on “society.”

**Table 1:** Identified studies focusing on rural areas classified as pre-pandemic.

No.	Authors (Year)	Country	NCD Condition	Summary	Results	Conclusion
[22]	Edwards R et al. (2000)	Tanzania	Hypertension	Compared prevalence and treatment status of hypertension in urban and rural areas. Hypertension was highly prevalent in rural areas, but awareness, treatment, and management rates were low. Cost-effective strategies for primary prevention, detection, and treatment were required.	Hypertension was highly prevalent, but awareness, treatment, and management rates were low.	Cost-effective strategies for prevention, detection, and treatment are required.
[11]	Cheng L et al. (2013)	China	Chronic diseases	Compared mortality data between urban and rural areas in Hubei Province. Chronic diseases were the leading cause of death, with higher mortality rates in rural areas.	Chronic diseases accounted for the majority of deaths, with higher rates in rural areas.	Strengthening NCD prevention strategies considering regional disparities is required.
[23]	Crampin AC et al. (2016)	Malawi	Hypertension, diabetes	Analyzed regional differences in hypertension and diabetes. Lifestyle-related diseases and obesity were increasing.	Participation in population health survey programs differed between urban and rural areas.	NCD interventions should be designed according to regional characteristics.
[24]	Kalkonde Y et al. (2019)	India	Chronic diseases	Long-term follow-up of causes of death in rural areas. NCDs became the leading cause of death, surpassing infectious diseases.	NCDs became the leading cause of death, exceeding infectious diseases.	Strengthening health systems to address chronic diseases is required.
[25]	Agyeman N et al. (2019)	Ghana	Dementia, chronic diseases	Examined understanding of dementia in rural areas. Dementia was perceived as part of aging, and family members provided care.	Dementia was perceived as aging, with family members providing care.	Institutionalization of elderly care policies is required.
[12]	Isangula KG et al. (2020)	Tanzania	Hypertension	Examined trust in physicians in hypertension care. Interpersonal behavior and technical competence influenced trust.	Interpersonal behavior and technical competence were key determinants of trust.	Improving trust is important for improving NCD care in rural areas.
[26]	Mukundiyukuri JP et al. (2020)	Rwanda	Chronic diseases	Evaluated availability, cost, and stockouts of essential NCDs medicines.	Medication availability was approximately 70%, but stockouts and high costs were challenges.	Collaboration between government and NGOs is essential for stable supply.
[13]	Nyaaba GN et al. (2020)	Ghana	Hypertension	Analyzed factors hindering hypertension management from the perspective of healthcare providers.	Lack of education, workforce shortages, and drug shortages contributed to poor management.	Expansion of training and strengthening of collaboration systems are required.
[27]	Odland ML et al. (2020)	Burkina Faso	Chronic diseases	Investigated multimorbidity among older adults and its impact.	Multimorbidity was observed in approximately 23% and was strongly associated with functional decline.	Prevention and integrated management of multimorbidity are required.
[28]	Singh KN et al. (2021)	Fiji	Obesity, chronic diseases	Investigated dietary factors associated with obesity among indigenous populations.	Food intake was influenced by multiple factors including sociocultural aspects.	Intervention design considering cultural context is required.
[29]	Kala S et al. (2021)	India	Chronic diseases	Surveyed behavioral and metabolic risk factors among adolescents.	Unhealthy diet and overweight were prevalent.	Early intervention for NCD prevention is required.
[30]	Meng S et al. (2022)	China	Diabetes, angina	Examined the know–do gap in chronic disease management among healthcare providers.	A gap between knowledge and practice was observed.	Quality evaluation and standardization are required.

Abbreviation: NCDs, non-communicable diseases.

**Table 2:** Identified studies focusing on rural areas classified as post-pandemic.

No.	Authors (Year)	Country	NCDs Condition	Summary	Results	Conclusion
[31]	Du YR et al. (2023)	China	Hypertension, diabetes, coronary heart disease, COPD	Compared NCDs prevalence among ethnic groups.	Differences in multimorbidity were observed across ethnic groups.	Interventions considering cultural and socioeconomic factors are required.
[32]	Singh U et al. (2023)	South Africa	Hypertension, diabetes	Examined unmet healthcare needs in patients with chronic diseases.	Approximately half had unmet healthcare needs.	Integrated care across diseases is required.
[33]	Samanthapudi AV et al. (2024)	India	Chronic diseases	Analyzed community participation in health promotion using photographic data.	Six themes were identified.	Community participation is important for health promotion.
[34]	Hosu MC et al. (2024)	South Africa	Chronic diseases	Examined comorbidities in drug-resistant tuberculosis and NCDs.	Comorbidities were associated with poor treatment outcomes.	Integrated management including NCDs is required.
[35]	Betz C et al. (2025)	Germany	Obesity, chronic diseases	Examined the influence of social determinants on weight loss.	Social determinants influenced weight loss processes.	Interventions incorporating social determinants are required.
[36]	Chakravartty M et al. (2025)	Bangladesh	Diabetes, hypertension	Examined anxiety and depression among patients with NCDs.	Anxiety and depression were prevalent.	Integration of mental health into NCD care is required.
[37]	Vanhoutte L et al. (2025)	Senegal	Chronic diseases	Reported a shift in causes of death from infectious diseases to NCDs.	Mortality from NCDs increased and surpassed that of infectious diseases.	Both prevention and treatment systems are required.
[38]	Kumari J et al. (2025)	India	Diabetes	Investigated diabetes prevalence.	The prevalence was 6.2%, with obesity as a major risk factor.	Prevention strategies focusing on lifestyle and education are required.
[39]	Debbarma S et al. (2025)	India	Hypertension, diabetes, etc.	Assessed medication adherence and determinants among rural Indian adults with NCDs.	Medication adherence was suboptimal and influenced by socioeconomic and healthcare factors.	Improving adherence requires targeted interventions addressing social and health-system barriers.
[40]	Mohandas NV et al. (2025)	India	Coronary artery disease	Investigated predictors of recurrent coronary events and secondary prevention.	Poor adherence and socioeconomic disadvantage were linked to recurrent events.	Strengthening secondary prevention may improve outcomes in coronary disease.
[41]	Hore TK et al. (2026)	Bangladesh	Hypertension, diabetes	Explored barriers and digital opportunities for NCD management.	Workforce shortages, resource constraints, and service gaps hindered NCD care.	Health-system strengthening and digital health may improve rural NCD management.

Abbreviation: NCDs, non-communicable diseases; COPD, chronic obstructive pulmonary disease.

## 4. Discussion

Among studies classified as pre-pandemic and post-pandemic, rural healthcare challenges related to NCDs were commonly reported. These included the increasing prevalence and risk of NCDs and low management rates, which were observed both before and after the pandemic. However, after the pandemic, additional societal dimensions emerged, including multimorbidity and complexity, diversity in cultural and behavioral characteristics, and the use of digital technologies. These findings suggest that comparatively different themes were reported among studies classified as pre-pandemic and post-pandemic. These findings may reflect greater attention to challenges related to NCDs within rural communities following the pandemic. In addition, the COVID-19 pandemic might have influenced the underlying burden, management, and healthcare context of NCDs in rural populations, which may also be reflected in the reported themes.

One possible reason for the greater focus on societal aspects in studies classified as post-pandemic is that community-level challenges became more visible after the COVID-19 pandemic. Infection control measures have led to the spread of remote work, behavioral restrictions, and the rapid introduction of digital technologies [7,42]. While these changes accelerated the adoption of online medical care and remote monitoring, they also revealed new challenges, including digital disparities, psychosocial health issues, and socioeconomic inequalities [43]. It is also possible that, in rural areas that were previously physically difficult to access, the introduction of digital technologies made diverse challenges more visible and enabled the selection of community-based approaches. The diversity of cultural and behavioral characteristics observed after the pandemic extends beyond digital literacy issues and includes socioeconomic disparities and ethnic and cultural practices [44].

In urban areas, NCD management had shifted toward integrated approaches, such as chronic disease management and community-based integrated care, before COVID-19 [45,46,47]. Although information and communication technology has been introduced into rural areas, implementing approaches similar to those in urban areas could remain difficult because of limitations in infrastructures and human resources [5,44]. While rural areas had utilized telemedicine, urban areas surpassed them after the pandemic, widening this gap [48]. Furthermore, some reports have indicated that digital health adoption has been limited among rural, as well as among low-income, and/or older populations owing to insufficient digital circumstance and literacy, thereby exacerbating regional disparities [10,42]. Thus, even though approaches to NCD management in urban and rural areas might have become more similar after the pandemic, there is concern that disparities may have widened. This suggests that rural areas may require approaches tailored to their specific contexts rather than the direct adoption of urban models. For example, community-based mutual support remains relatively present in rural areas [49]. From a community perspective, strategies utilizing resources unique to rural areas are expected to be important.

This study had several limitations. First, the search strategy focused on specific keywords, and studies discussing challenges without using the term “challenge” or “issue” may not have been included in the review. Second, the number of included articles was limited, possibly because few studies explicitly focused on pandemic-related challenges. Third, differences in culture, economy, and healthcare systems across rural areas worldwide limit the generalizability of the findings. Fourth, this study used a qualitative approach, and the quantitative changes were not assessed. Fifth, the observation period was relatively short in this study. Further studies considering long-term impacts are needed. In addition, although studies were classified using the best available information, including publication period, study period, data collection period, submission dates, and explicit discussion of COVID-19-related effects, some uncertainty remains regarding the distinction between pre-pandemic and post-pandemic studies. Therefore, findings related to themes identified in the two groups should be interpreted carefully.

## 5. Conclusions

This study examined the reported rural healthcare challenges related to NCDs before and after the COVID-19 pandemic and summarized themes reported in the literature. Studies classified as pre-pandemic primarily focused on healthcare-related aspects, whereas studies classified as post-pandemic showed a greater focus on broader societal aspects. The COVID-19 pandemic might have brought greater attention to challenges within rural areas. Further research is required to clarify the significance and implications of these reported themes in rural healthcare.

## Data Availability

No new data were created or analyzed in this study.

## Supplementary Materials

The following supporting information can be downloaded at: https://www.mdpi.com/article/10.3390/ijerph23080971/s1, File S1: Preferred Reporting Items for Systematic reviews and Meta-Analyses extension for Scoping Reviews (PRISMA-ScR) Checklist.
